# Recent Progress in Silicon−Based Materials for Performance−Enhanced Lithium−Ion Batteries

**DOI:** 10.3390/molecules28052079

**Published:** 2023-02-22

**Authors:** Xiangzhong Kong, Ziyang Xi, Linqing Wang, Yuheng Zhou, Yong Liu, Lihua Wang, Shi Li, Xi Chen, Zhongmin Wan

**Affiliations:** 1 Hunan Institute of Science and Technology, College of Mechanical Engineering, Yueyang 414006, China; 2 Hunan Institute of Science and Technology, Institute of New Energy, Yueyang 414006, China

**Keywords:** Si—based materials, anode, modification strategy, lithiation/de−lithiation mechanism, lithium−ion batteries

## Abstract

Silicon (Si) has been considered to be one of the most promising anode materials for high energy density lithium−ion batteries (LIBs) due to its high theoretical capacity, low discharge platform, abundant raw materials and environmental friendliness. However, the large volume changes, unstable solid electrolyte interphase (SEI) formation during cycling and intrinsic low conductivity of Si hinder its practical applications. Various modification strategies have been widely developed to enhance the lithium storage properties of Si−based anodes, including cycling stability and rate capabilities. In this review, recent modification methods to suppress structural collapse and electric conductivity are summarized in terms of structural design, oxide complexing and Si alloys, etc. Moreover, other performance enhancement factors, such as pre−lithiation, surface engineering and binders are briefly discussed. The mechanisms behind the performance enhancement of various Si−based composites characterized by in/ex situ techniques are also reviewed. Finally, we briefly highlight the existing challenges and future development prospects of Si−based anode materials.

## 1. Introduction

In recent years, lithium−ion batteries (LIBs) have successfully dominated the power source fields including portable electronic devices, electric vehicles (EV) and large−scale storage systems because of their high capacity, long lifespan and environmental friendliness [1,2]. However, with the rapidly increasing demands of energy markets, it is urgent to develop next generation LIBs with higher energy and power densities. Commercial anode materials such as graphite cannot meet requirements such as much higher energy/power densities and long cycle life because of its limited theoretical capacity (372 mAh g^−1^) and low energy density (~150 Wh kg^−1^). For a long time, researchers have been devoted to exploiting advanced anodes with high abundance, low cost and excellent lithium−ion storage properties. Numerous anode materials for LIBs have been studied, such as Li_4_Ti_5_O_12_, transition metal oxides (MnO, Nb_2_O_5_, NiO et al.), transition metal sulfides (Ni_2_S_3_, MoS_2_, VS_2_ et al.) and alloys (Sb, Sn). Among them, silicon (Si) has attracted increasing attention recently as one of the most likely alternatives to graphite.

**Figure 1:**
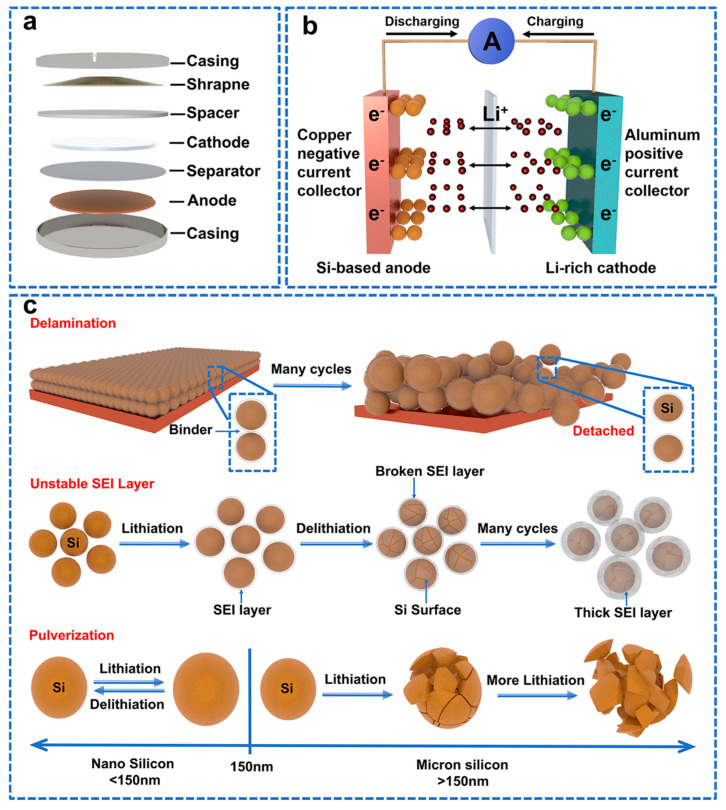
(**a**) Diagram of lithium-ion button battery. (**b**) Schematic diagram of lithium−ion Si−anode battery. (**c**) Three representative failure mechanisms of Si anodes: delamination, unstable SEI layer, and pulverization [24].

The highest theoretical capacity of Si (4200 mAh g^−1^, 10 times than graphite) is due to the fact that one Si atom can bond with approximately four lithium ions (Li_4.4_Si) [3]. Moreover, the lithiation potential of Si relative to Li/Li^+^ is 0.4 V, higher than that of graphite (about 0.05 V), hindering the formation of lithium plating and lithium dendrites [4]. Importantly, Si has the second−largest reserve on earth (exceeding 28% of the earth’s mass) and can be widely available in nature in silicate mineral, solar panels, rice husks, straw, bamboo stalks and sugar cane. Most of above−mentioned resources can hardly be recycled with high added value or low environmental pollution. Therefore, developing strategies to utilize Si resources in energy storage systems has significant value for the advanced electrodes of next−generation LIBs and green sustainable development [5]. Figure 1a,b show the schematic diagram of a lithium−ion Si−anode battery [6]. Charge and discharge are carried out through lithiation and de−lithiation processes of electrochemical reactions. Lithium ions spread back and forth through an electrolyte between the anode and cathode. In the lithiation process, the metal lithium oxide at the cathode breaks down to produce lithium ions, which store electricity by combining electrons with the material at the negative electrode through an electrolyte. In the process of de−lithiation, the lithium bonded to the anode breaks down to produce lithium ions and electrons, which move to the anode and combine with the metal lithium oxide, generating electrons to power the device.

Unfortunately, unavoidable serious drawbacks still restrict the commercial applications of Si. As shown in Figure 1c, there is huge volume expansion (>300%) of Si during lithiation. Volume expansion was confirmed by imaging techniques and recording of different Li –Si phases [7]. This volume expansion increases the stress on the Si, leading to Si powder. Moreover, volume expansion will push the conductive material on the active material away from its surface, resulting in low electrochemical performance. During the first lithiation process, SEI is formed on the surface of the silicon electrode through the decomposition of the electrolyte [8]. SEI allows lithium−ion conduction but does not allow electron flow, which limits electrolyte consumption and improves the cycling performance of the lithium−ion battery. The electrochemical performance of lithium−ion batteries is highly dependent on SEI quality [9]. However, due to the volume expansion of Si, the organics in the electrolyte decompose to form an unstable SEI. The unstable SEI can be easily broken and re−formed on the Si surface, which continuously consumes large amounts of lithium ions, reduces the initial Coulombic efficiency (ICE) and eventually depletes the electrolyte [10,11]. The formation of these defects will be discussed in detail in the mechanism section. Apart from defects during cycling, Si is a semiconductor material with poor conductivity in a metastable structure (10^−3^ S cm^−1^ 25 °C) [12].

Researchers have aspired to solve and mitigate these problems. These strategies include Si nanoparticles [13,14], nanowires [15] and nanotubes. To further alleviate the volume expansion of Si—based materials during lithiation, Si electrode composites have been designed, such as Si/C composites [16,17], transition−metal oxide composites [18] and Si alloy composites [19]. Another way to address these challenges is to improve electrolytes and adhesives. The introduction of appropriate additives contributes to the formation of thin and stable SEI layers, reducing the consumption of additional electrolyte during the cycle and improving cycle stability [20].

Shih et al. prepared 3D sphere−like Si/graphite by combining electrostatic self−assembly and spray−drying methods. Due to the structural stability of the microsphere particles and the effective use of the graphite buffer layer, the discharge capacity increased from 764 to 1210 mAh g^−1^ after 30 cycles at a current density of 0.1A g^−1^ compared to bare Si/G [21]. Liu et al. further combined silicon from spent solar cells with CP substrate through a ball−milling process in order to form Si/SiO_x_/Al_2_O_3_−CP composite electrodes [22]. Among the ball−milled samples, two hours of ball milling at 500 rpm showed the highest initial discharge capacity of 4340 mAh g^−1^. Zhang et al. combined ball−milling and spray−drying methods to prepare watermelon−shaped internally expanded core–shell buffer structured Si/electrochemically exfoliated graphene/C (Si/EG/C) composites [23]. EG not only has good electrical conductivity, but also compresses (lithiation process) or expands (de−lithiation process) between the silicon particles and the strong carbon shell to control the volume change inside the Si/EG/C particles, thus maintaining the stability of the SEI film and readily obtaining good electrochemical properties. The Si/EG/C anode exhibits a capacity retention of approximately 78.3% after 500 cycles. The discharge capacity retention of a full cell with LiCoO_2_ after 500 cycles is almost 100% of the initial discharge capacity (160 mAh g^−1^) after 500 cycles.

This review starts with Si and summarizes recent advances in architecture design of Si, Si alloy structure control, and SiO_x_ and composite structure of Si/Si oxide. Second, the effects of surface engineering, pre−lithiation, and binders on the electrochemical performance of Si anode materials are described. Then, the latest characterization techniques of Si—based materials, structural control of Si–graphene composites, SEI formation mechanisms and optimal surface oxide layers are introduced. Finally, the existing problems and future development prospects of Si−based anode materials are briefly introduced.

## 2. Silicon−Based Material Modification Strategies

### 2.1. Micro/Nanometer Architecture Design

**Figure 2:**
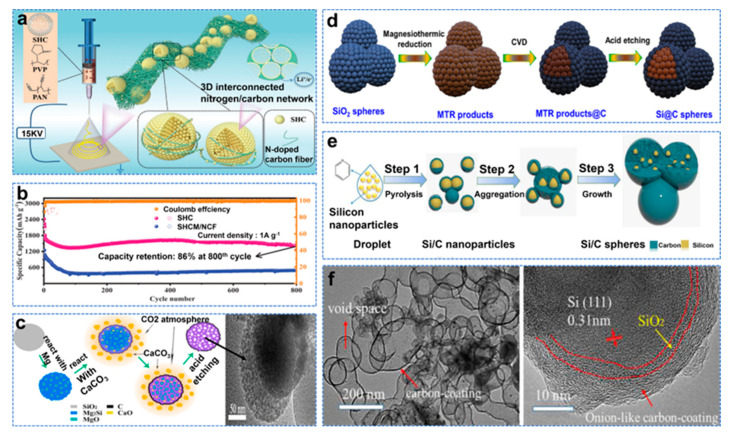
(**a**) Schematic illustration of formation procedure of the SHCM/NCF paper, (**b**) cycling property at 1 A g^−1^ [30]. (**c**) Schematic illustration of the reaction from SiO_2_ sphere to the final Si@C composite and TEM image of Si@C composite [31]. (**d**) Schematic illustration of the formation process of the composite Si anode [33]. (**e**) Schematic illustration of the formation of onion−like Si/C spheres, (**f**) TEM and HRTEM image of sample [34].

The nanostructure strategy is to reduce the size of Si particles to nanometer level (<150 nm), increase the contact area between particles and reduce the problem of Si anode volume expansion to improve long cycle performance. However, large specific surface areas lead to low initial Coulombic efficiency (ICE) [25]. Moreover, the change in electrode structure during the cycle can easily affect electron transport [26]. The carbon layer can slow down the volume change of the Si anode electrode, improve electrical conductivity, and continuously form a stable SEI [27]. In addition, the hollow structure of carbon can accommodate a large number of active nanodots to further improve electrochemical performance [28]. Electrospinning is a new technique to produce continuous mesh structure at the nano scale, which is a convenient and excellent method for preparing Si electrodes [29]. Zhu et al. used self−assembled microspheres and interwoven fabrics to prepare linked hollow carbon nanospheres made of 3D interconnected nitrogen/carbon networks with uniformly distributed Si nanodots (SHCM/NCF), as shown in Figure 2a [30]. The reversible capacity was 1442 mAh g^−1^ at 800 cycles of 1 A g^−1^ in a half cell, as shown in Figure 2b. However, the Si nanostructure and carbon nanocoating are usually formed in separation, making the whole synthesis process costly, complex and time−consuming. Huang et al. proposed converting Si nanomaterials (diatomite and Si spheres) into Mg_2_Si [31]. After the further reaction of the transformed Mg_2_Si and CaCO_3_, a conformal carbon nanolayer of 1–5 nm spontaneously grows on the newly formed Si nanostructure, and Si@C is obtained, as shown in Figure 2c. Si@C provides a reversible capacity of 1359.7 mAh g^−1^, and 764.6 mAh g^−1^ was retained after 500 cycles at 4 A g^−1^. The products of thermal reduction of magnesium in the above method (including MgO, Mg_2_Si, unreacted Mg and SiO_2_) are usually removed by HF. This method is dangerous and harmful to the environment. Mg contains unique catalytic activity, which can be used as a catalyst for in situ growth and preservation of the carbon layer of Si particles [32]. Zhao et al. encapsulated Si nanoparticles by growing graphite carbon with chemical vapor deposition (CVD) [33]. The by−products of the magnesium thermal reduction reaction were used as templates and catalysts to form a three−dimensional conductive network structure, as shown in Figure 2d. It demonstrated excellent lithium storage properties with high specific capacity (2126 mAh g^−1^, 0.1A g^−1^) and an extraordinary rate capability (750 mAh g^−1^, 5 A g^−1^). However, due to the presence of SiO_2_, its capacity decays rapidly, but the carbon coating with a unique onion structure has high structural stability. The onion structure can effectively buffer Si volume expansion, which is more conducive to the long life of LIBs. Wang et al. designed a Si/C composite material similar to the onion with pyridine as a carbon source, as shown in Figure 2e, whose TEM and HRTEM images are shown in Figure 2f. As the anode material of a lithium−ion battery, the composite material circulates 400 cycles at 0.2 A g^−1^ current density and has a capacity up to 1391 mAh g^−1^ [34].

**Figure 3:**
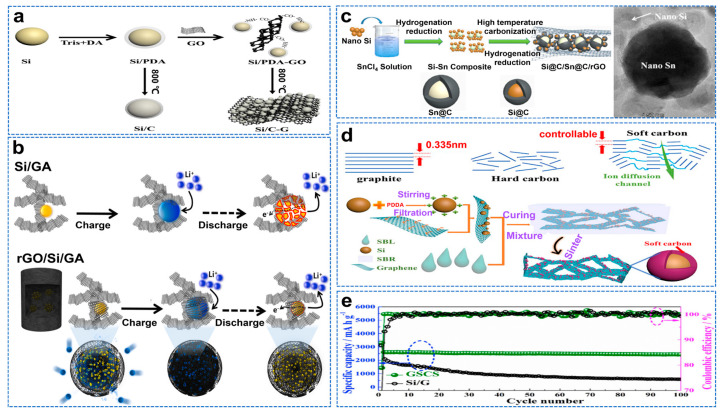
(**a**) Preparation of Si/C−G materials [38]. (**b**) Si/GA and rGO/Si/GA composite anodes before and after lithiation/de−lithiation processes [40]. (**c**) Schematic illustration of the synthesis of Si@C/Sn@C/rGO and TEM images of sample [41]. (**d**) Schematic of preparation of GSCS. (**e**) Cycling performance of GSCS and Si/G at 0.2 A g^−1^ [42].

Graphene oxide (GO) is a derivative and precursor of graphene, rich in oxygen−containing functional groups [35]. It is known for its good hydrophilicity and processing properties. Therefore, graphene oxide can be used to study the anode materials of lithium−ion batteries combined with nano−Si [36]. The self−polymerization of dopamine can provide multiple secondary amine groups and form strong hydrogen bonds with the carboxyl groups on the surface of GO through chemical cross−linking, and dopamine also provides a nitrogen source, providing additional active potential [37]. Han et al. used dopamine as a carbon source to wrap Si particles and load them in the gap space of the graphene sheet, as shown in Figure 3a [38]. The Si/C−G anode material exhibits a high reversible capacity of 1196.1 mAh g^−1^ after 700 cycles at 357.9 mAh g^−1^. Graphene aerogel (GAs) have high electrical conductivity and mechanical stability, evenly disperse Si nanoparticles, promote the rapid and effective diffusion of Li^+^, and inhibit volume expansion [39]. Kim et al. fabricated reduced graphene oxide (rGO) and Si nanoparticles into spherical composite structures using a facile spray−drying process [40]. The microspheres were uniformly incorporated into a 3D porous graphene aerogel (GA) structure using an aerogel synthesis process. This multilayer encapsulation structure relieves volume expansion and achieves superior rate performance (819 mAh g^−1^ at 10 C). Figure 3b is a schematic diagram of the lithiation and de−lithiation process of the Si/GA and rGO/Si/GA anode composites. As the active matrix of Si, Sn has high ductility and conductivity. Zhu et al. prepared dense arrays of Si@C and Sn@C nanoparticles anchored by GO (denoted Si@C/Sn@C/rGO) by solution impregnation and hydrogenation reduction, as shown in Figure 3c [41]. The prepared Si@C/Sn@C/rGO electrode has improved ICE (78%), rate capacity and significantly enhanced cycle stability (a high reversible capacity of nearly 1000 mAh g^−1^ can be obtained after 300 cycles at a current density of 1 A g^−1^). The disordered microstructure of hard carbon leads to low conductivity and hinders Si alloy reaction, while the encapsulation of the soft carbon layer reduces volume expansion and has alternating amorphous and crystalline regions, which can provide a fast diffusion channel for lithium ions. Wang et al. encapsulated nano−Si in soft carbon and embedded it in a graphene frame to form a three−dimensional independent composite material, as shown in Figure 3d [42]. As a result, the composite anode has a superior specific capacity, cycling performance and rate performance of about 2600 mAh g^−1^ at 0.2 A g^−1^. After 100 cycles, there is almost no capacity attenuation, as shown in Figure 3e.

**Figure 4:**
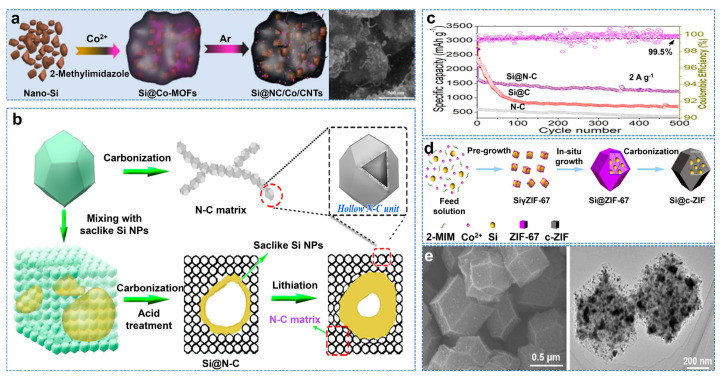
(**a**) Schematic illustration of the synthesis process for the Si@NC/Co/CNT composites and typical SEM images of sample [46]. (**b**) Schematic illustration of the fabrication of Si@N−C composite. (**c**) Cycle performance of Si@N−C, Si@C, and N−C at 2 A g^−1^ [48]. (**d**) Schematic illustration of the synthesis of Si@c−ZIF. (**e**) TEM and HRTEM image of sample [49].

Porous carbon frames often expose Si to the surface, resulting in unstable SEI [43]. Due to the excellent impermeability of graphene, it may hinder the diffusion of lithium ions. Therefore, as a carbon layer, a metal–organic skeleton (MOF) can effectively encapsulate Si and form a 3D frame structure, which has been proven to be the ideal carbon frame for lithium batteries [44,45]. Luo et al. embedded Si nanoparticles into the porous framework of nitrogen−doped carbon/cobalt/CNTs (Si@NC/Co/CNTs) and synthesized Si matrix composites, as shown in Figure 4a [46]. Si@NC/Co/CNTs have excellent cycling performance (758 mAh g^−1^ after 800 cycles at 1 A g^−1^). The composites prepared with Zn−MOFs have larger specific surface areas than those prepared with Co−MOFs. Moreover, ZIF−8 containing N and Zn ions can be transformed into ZnO/N−doped carbon composites or nitrogen−doped carbon composites at different temperatures [47]. Therefore, ZIF−8 has excellent potential in Si/C composites due to its controllable configuration and high surface area. Wei et al. also used the molten−salt magnesium thermal reduction method to prepare spongy carbon−based anchored saccular Si using ZIF−8 as raw material, as shown in Figure 4b [48]. The saccular Si nanoparticles and the buffer space in the sponge matrix can coordinate to adapt to the volume change of Si and maintain the integrity of the electrode. At 0.2 A g^−1^, the initial Coulombic efficiency is 84%. After 500 cycles, the capacity of the composites is 1448 mAh g^−1^ at 2 A g^−1^, as shown in Figure 4c. Gao et al. prepared Si@C−ZIF by in situ encapsulation of Si nanoparticles to form a metal–organic skeleton (MOF)−derived carbon shell, as shown in Figure 4d, whose TEM and HRTEM images are shown in Figure 4e [49]. It exhibits excellent electrochemical properties, with a capacity of 3714 mAh g^−1^ at 0.2 A g^−1^ and a reversible capacity of 820 mAh g^−1^ at 5 A g^−1^, even after 1000 cycles.

Nano−Si/C composites, with nano−Si as the core to provide capacity and the carbon shell as the conductive framework, can limit the volume expansion of nano−Si to provide good electrochemical stability. However, the reduction of the nano−Si content of the composite material will inevitably lead to the reduction of its capacity, so it is very necessary to explore the ratio of nano−Si and C that can ensure the capacity. Embedding nano−Si into graphene can further improve the electronic conductivity and avoid the aggregation of nano−Si particles. However, graphene cannot fully coat nano−Si particles, and the exposed nano−Si surface will come into direct contact with the electrolyte, forming an unstable SEI, thereby degrading its electrochemical performance. The three−dimensional MOF structure can encapsulate Si nanoparticles well, avoid the direct exposure of nano−Si particles to the electrolyte, and ensure the stability of their electrochemical performance. However, three−dimensional structures are fragile, so elastic three−dimensional frameworks may be more suitable for nano−Si particles.

### 2.2. Si Alloy Structure Control

**Figure 5:**
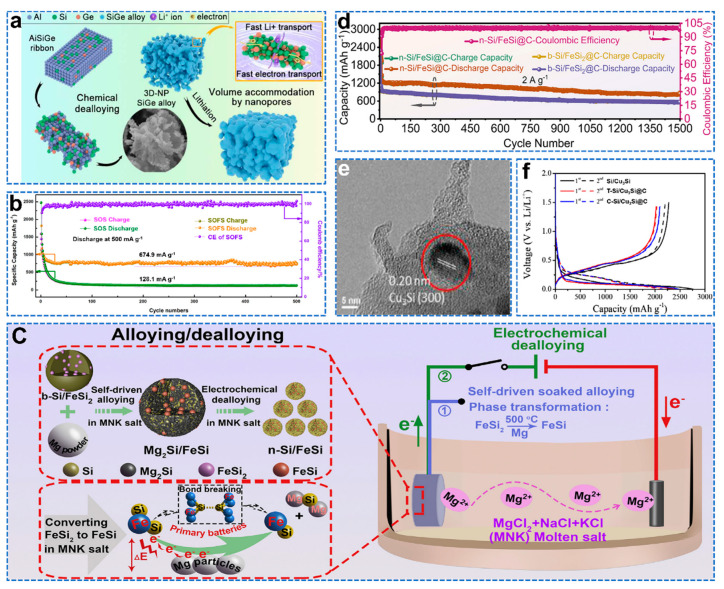
(**a**) Schematic of the evolution of 3D−NP SiGe structure via chemical dealloying [51]. (**b**) The galvanostatic cycle performance of SOS and SOFS electrodes at 500 mA g^−1^ [55]. (**c**) Schematic illustration of the self−driven alloying–electrochemical dealloying process for the formation of n−Si/FeSi. (**d**) Cycle performance at 2 A g^−1^ [56]. (**e**) Magnified TEM image of red circles. (**f**) Galvanostatic lithiation and de−lithiation voltage profile of Si/Cu_3_Si, T−Si/Cu_3_Si@C and C−Si/Cu_3_Si@C conducted at 0.2 A g^−1^ [57].

Si alloy reduces internal mechanical stress and improves electrical conductivity compared to pure Si [50]. Yang et al. successfully prepared three−dimensional nanoporous (3D−NP) SiGe alloys by dealloying using AlSiGe alloys as precursors, and controlled their morphology and pore size by adjusting the Al content, as shown in Figure 5a [51]. It has better ICE and better rate performance due to the accelerated rate of lithium−ion transport by Ge and the different starting potentials for the reaction of Si and Ge with Li, which allows for a gradual release of strain. NbSi_2_ can be used as a buffer matrix to facilitate electrochemical reactions and effectively slow down the volume expansion of composites [52]. Bae et al. prepared carbon−free Nb/NbSi_2_@Nb_2_O_5_ composite materials from SiO and Nb through a high−energy mechanical milling process [53]. While silicon alloys have good electrical conductivity, this inevitably reduces capacity and increases the cost of the anode, making silicon alloys more attractive as composites with silicon. TiSi_2_ not only acts as a buffer for the volume expansion of Si, but also improves electrical conductivity. Zhang et al. prepared Si/TiSi_2_ from low−cost PV and metallurgical scrap by melting and ball−milling in a scalable manner to obtain high specific capacity [54]. Qiu et al. formed new structures of Si/FeSi_2_ nanoparticles protected by SiO_x_ shells through passivation (SOFS) [55]. The presence of SiO_x_ promotes the formation of a stable SEI, while FeSi_2_ acts as a buffer phase to improve the electrochemical stability of the electrode, resulting in good stability, as shown in Figure 5b. Ma et al. prepared nano Si/FeSi by electrochemical dealloying of an industrial bulk ferrosilicon alloy (b−Si/FeSi_2_), as shown in Figure 5c [56]. A polydopamine−derived carbon coating was then used to further enhance the storage properties of the lithium. The n−Si/FeSi@C anode exhibits a high capacity of 1449.7 mAh g^−1^ after 500 cycles at 0.4 A g^−1^ and maintains an extremely stable reversible capacity of 846.8 mAh g^−1^ even after 1500 cycles at 2 A g^−1^, as shown in Figure 5d. Wu et al. prepared micron−scale C−Si/Cu_3_Si@C by self−assembly of Si/Cu_3_Si composite with varying submicron sizes using dopamine as the first carbon precursor and ethylene (C_2_H_4_) as the second carbon precursor [57]. For Cu_3_Si, the d−spacing of the stripes is 0.20 nm, corresponding to the (300) plane, as shown in Figure 5e. Therefore, the C−Si/Cu_3_Si@C composite performs best under stable cycling conditions. The de−lithiation capacity of the first cycle is 2101 mAh g^−1^, and the initial Coulombic efficiency is 84.7%, as shown in Figure 5f. Compared to Si, Si alloys offer better electrical conductivity and less volume expansion. However, silicon alloys inevitably reduce the capacity of the anode and increase the cost. Therefore, Si alloy/Si composites not only reduce the cost but also achieve a larger specific capacity, allowing for better electrochemical stability when compounded with carbon frameworks and oxides.

### 2.3. SiO_x_ (0 < x < 2)

**Figure 6:**
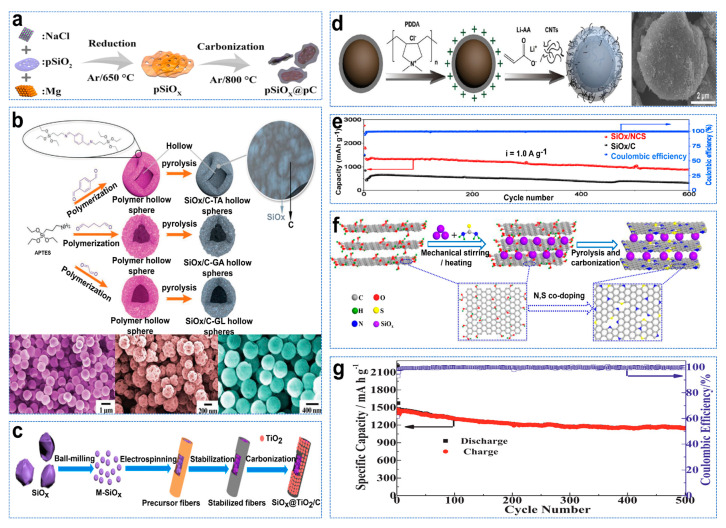
(**a**) Schematic illustration of the synthesis of pSiO_x_@pC [59]. (**b**) Schematic illustration of the synthetic procedure for SiO_x_/C HS−TA, SiO_x_/C HS−GA, SiO_x_/C HS−GL and SEM images [60]. (**c**) Schematic of preparation of the SiO_x_@TiO_2_/C fibers [62]. (**d**) Schematic illustration of the preparation of C−SiO_x_/C and SEM images [63]. (**e**) Long−term cycling performances of SiO_x_/NCS and SiO_x_/C at 1 A g^−1^ [66]. (**f**) Schematic illustration of the fabrication process of the SiO_x_/N/S−rGO composite. (**g**) Cycling performance at 0.5 A g^−1^ [68].

The theoretical capacity of SiO_x_ (2200–3580 mAh g^−1^) is lower than that of Si. However, the inert electrochemical products of Li_2_O and Li_4_SiO_4_ are generated during the first lithium−ion process of an SiO_x_ anode, which can be used as a buffer matrix to form a stable SEI layer. It helps to adapt to the significant volume changes in subsequent lithium processes, thus extending the cycle life [58]. Although the volume variation of micrometer−sized SiO_x_ particles (≈200%) is smaller than that of Si (≈400%), its inherent conductivity is poor. Carbon coating on the surface of SiO_x_ is one of the most effective methods to improve its electronic conductivity. The carbon matrix coated on the surface of SiO_x_ provides a rigid frame for SiO_x_ to mitigate its volume changes during circulation. Ge et al. constructed and prepared pSiO_x_@pC composite material with a 3D network, as shown in Figure 6a [59]. Because porous SiO_x_ can also accommodate its volume expansion during the lithiation process, improve its structural stability and shorten the transmission length of lithium ion from the electrolyte to electrode, it has excellent electrochemical performance. The hollow structure can prevent volume expansion well. Zhou et al. used 3−aminopropyltriethoxysilane (APTES) and dialdehyde molecules as precursors of Si and carbon to produce polymer hollow spheres (PHSs) through one−step condensation of aldehydes [60]. Then, in situ pyrolysis of PHS ensured SiO_x_ was uniformly doped into the carbon hollow capsid at the nanocluster scale, as shown in Figure 6b. TiO_2_ has a small volume change (<4%), with strong mechanical stability; stable SEI film can be formed, and lithium TiO_2_ can also improve electronic conductivity and lithium−ion diffusion [61]. Tan et al. successfully prepared SiO_x_@TiO_2_/C fibers with a TiO_2_/C composite layer using the electrostatic spinning method, as shown in Figure 6c [62]. Li et al. stabilized the electrochemical performance of SiO_x_ by constructing a dynamic and stable interface composed of polyacrylate nanolayers and multi−walled carbon nanotubes on carbon−coated SiO_x_ particles, as shown in Figure 6d [63]. Homogeneous incorporation of CNTs at the Li−PAA interface provided rapid electronic channels that were electrically conductive, ensuring excellent electronic conductivity of the composite particles. Graphene is a two−dimensional carbon material with a large surface area, good electronic conductivity, good chemical stability, good flexibility and other advantages. It can be used as a buffer matrix when paired with SiO_x_, effectively adapting to the volume change of SiO_x_ during the charge and discharge process [64]. At the same time, N−doping can improve the electrochemical reactivity and conductivity of graphene and promote charge transfer to provide more active sites [65]. Zhang et al. developed a simple and scalable synthesis method for SiO_x_ (SiO_x_/NCS) supported on nitrogen–oxide−doped carbon nanosheets by melamine−assisted ball−milling and annealing processes [66]. SiO_x_/NCS anodes exhibit ultra−stable cycle performance (~900 mAh g^−1^ over 600 cycles, 1 A g^−1^), as shown in Figure 6e. The addition of sulfur can also tune electrical properties, improve wettability, reduce charge transfer resistance, and improve electrochemical performance [67]. Shi et al. constructed a novel SiO_x_ anode material by embedding SiO_x_ particles into three−dimensional multilayer graphene flakes and co−doping them with N and S, as shown in Figure 6f [68]. In addition, first−principles calculations confirmed the synergistic effect of N and S co−doping, which introduced more heteroatomic defects and reduced bandgap, increasing negative adsorption energy of Li^+^, which further promoted electron transfer and improved storage capacity of Li^+^. After 600 cycles, the prepared anode material had a high reversible capacity of 1150 mAh g^−1^, as shown in Figure 6g.

Carbon−coated SiO_x_ can alleviate its volume expansion, improve electrical conductivity, and help improve ICE, while the low specific surface area alleviates side reactions. The graphene–oxide−wrapped SiO_x_ particles are uniformly anchored inside and on the surface of the graphite, improving mechanical flexibility and electrical conductivity. However, SiO_x_ itself has a low capacity, and its use in composite materials reduces the SiO_x_ content again, resulting in a lower capacity of these composite material. Therefore, the content of SiO_x_ in different SiO_x_ composite materials is very important, and it is worth continuing to explore.

### 2.4. Composite Structure of Si/Si Oxide

**Figure 7:**
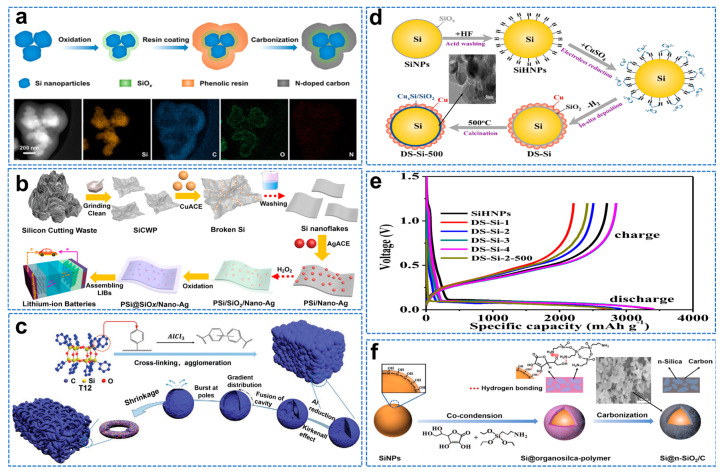
(**a**) Schematic illustration of the synthesis of Si@SiO_x_@C and EDS mappings [69]. (**b**) Illustration of the fabrication of Si@SiO_x_/Nano−Ag composite [70]. (**c**) The schematic illustration of synthesis of Si/SiO_x_/C nanorings [71]. (**d**) Schematic diagram of the synthetic process for DS−Si composites and SEM image. (**e**) First discharge/charge curves at 420 mA g^−1^ [73]. (**f**) Schematic illustration of the synthesis process for Si@n−SiO_2_/C composite and SEM image [74].

The interface interaction between the carbon layer and the Si surface is weak, and the continuous volume change will cause the cracking of the coating, while the separation of Si and carbon will lead to the constant formation of SEI film and ultimately lead to the deterioration of the cycling performance [25]. Hu et al. introduced SiO_x_ layers onto Si nanoparticles by annealing in the air to facilitate subsequent conformal carbon coatings [69]. Phenolic resin can be easily coated on SiO_x_ modified Si nanoparticles and converted into a conformal carbon coating shell through solid hydrogen bonding. After further carbonization, clear core–double−shell structured Si@SiO_x_@C can be obtained, as shown in Figure 7a. From the perspective of environment and economy, Xi et al. prepared pSi@SiO_x_/Nano−Ag composites by using photovoltaic Si cut waste (SiCW) economically and simply, as shown in Figure 7b [70]. The reversible capacity after 500 cycles is 1409 mAh g^−1^, which has a broad market prospect in the lithium−ion battery industry. Lin et al. used molten−salt−assisted low−temperature aluminothermic reduction of dodecaphenyl cage silsesquioxane (T_12_−Ph) to form hollow spheres via the Kirkendall effect [71]. As the reduction time increases, the hollow sphere surface collapses at the poles, eventually forming Si/SiO_x_/C nanorings. Finally, when the mass ratio of T_12_−Ph to AlCl_3_ is 1:10, held for 24 h at 300 °C, the Si/SiO_x_/C nanorings with the most uniform element distribution and the most significant ring structure are obtained. Si/SiO_x_/C nanorings with the most prominent structure are shown in the Figure 7c. Due to its special three−dimensional structure, it can still maintain a high reversible capacity of 520.7 mAh g^−1^ for 1000 cycles at 5 A g^−1^, with a capacity retention rate of 86%, and it can still maintain a high capacity of 517.9 mAh g^−1^ at the harsh temperature of −70 °C.

SiO_2_ acts as an adhesion coating to improve the interfacial adhesion between Si and carbon and forms irreversible products during the first lithiation to reduce volume expansion [72]. Ren et al. prepared multi−shell (Cu@Cu_x_Si/SiO_2_) coated Si (DS−Si) nanocomposites by in situ chemical deposition using Si particles as raw materials and copper sulphate as a copper source, as shown in Figure 7d [73]. The initial Coulomb efficiency of DS−Si composite is 86.2%, as shown in Figure 7e. However, the process is complex, energy−intensive and environmentally unfriendly. In addition, the continuous dense silica layer tends to produce stress concentration and crush the coating during discharge/charging. Dai [74] et al. successfully synthesized multifunctional crosslinked nano Si/carbon hybrid matrix (Si@n−SiO_2_/C) composites coated with Si nanoparticles by synchronous polycondensation of APTES with L−Ascorbic Acid (L−AA), as shown in Figure 7f.

SiO_x_ can provide higher capacity than carbonaceous materials and can effectively alleviate volume expansion, and the use of a SiO_x_ shell helps to form a stable SEI during cycling. However, the SiO_2_ outer layer can react with lithium, accompanied by the formation of poor ionic conductors, resulting in a decrease in its rate capability with increasing SiO_2_ layer thickness. In addition, a small amount of SiO_2_ shell cannot effectively alleviate volume expansion. Therefore, an optimal SiO_2_ layer thickness that can significantly alleviate volume expansion and exhibit the best performance is required.

## 3. Advanced Modification Strategies for Si−Based Cells

### 3.1. Pre−Lithiation

**Figure 8:**
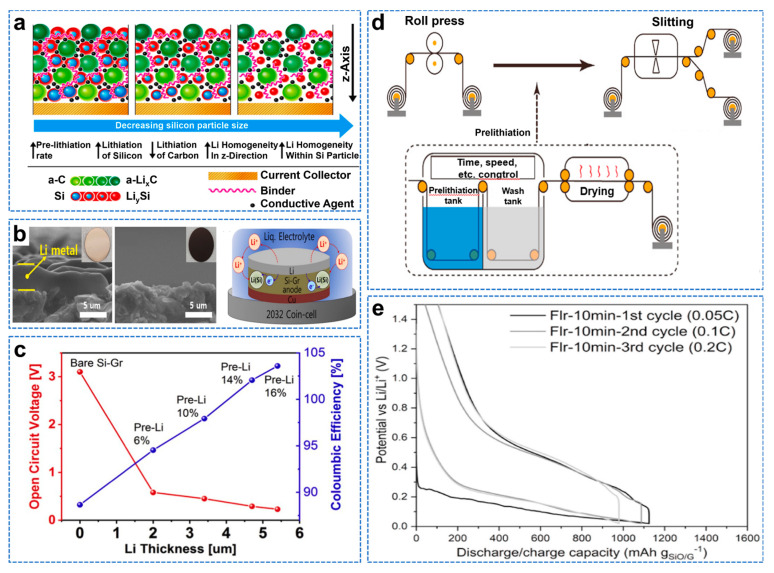
(**a**) Schematic illustration of the impact of Si particle size on the pre−lithiation behavior of Si/C composite anode materials in LIB cells, dependent on the Si particle size and electrode thickness (z−direction) [77]. (**b**) SEM images of the Li−evaporated Si−Gr anode, pre−lithiation Si−Gr anode and schematic representation of the pre−lithiation reaction process, (**c**) The open circuit voltages and the Coulombic efficiencies of the pre−lithiation Si−Gr anodes at different evaporated Li^+^ thicknesses [79]. (**d**) Schematic diagram of SiO pre−lithiation, (**e**) Lithiation/de−lithiation curves of the cell made with Flr−10min half−cell during formation process [81].

A battery will have the problem of capacity attenuation, and the initial irreversible loss will be generated at the first charge, when the electrolyte is decomposed on the anode surface to form SEI film [75]. Therefore, forming a continuous and stable SEI film can prevent intercalation and allow Li^+^ to pass through, thus improving the electrochemical performance of the battery. Storage of additional lithium in the anode electrode using pre−lithiation can significantly improve the initial Coulombic efficiency (ICE) [76]. However, the pre−lithiation process is complex; the battery production steps need to be simplified; and a more straightforward method of pre−lithiation is needed. A Si@C electrode can effectively alleviate volume expansion and improve electrochemical performance, while Si particle size affects the performance and the pre−lithiation. Barmann et al. studied the pre−lithiation capacity, pre−lithiation uniformity, and spatial distribution of Si@C anodes with different particle sizes, as shown in Figure 8a [77]. Si–graphite hybrid (Si–GR) electrodes have been shown to improve cycling performance. In addition, the degree of volume expansion can be controlled by controlling the Si/Gr mixing ratio. The volume change of Si can be inhibited by changing the pre−lithiation state of the Si−GR anode without reducing the size of Si particles or changing the morphology [78]. Kim et al. proposed a simple method of pre−lithiation by vaporizing lithium on a Si graphite anode with a diameter of 100 nm, as shown in Figure 8b [79]. Precisely controlling the thickness of pre−deposited lithium can effectively reduce volume expansion and improve the electrochemical performance, as shown in Figure 8c. Organic lithium compounds have a slightly higher potential of 0.3–0.5 V (vs. Li/Li^+^), preventing rapid and effective SiO anodes pre−deposition [80]. Zhang et al. reported a novel organic lithium compound (Li−9,9−dimethyl−9H−fluorene−tetrahydrofuran) with a low redox potential (~0.2 V) for the pre−lithiation of SiO based anodes, as shown in Figure 8d [81]. The SEI layer is formed during preloading, and a controllable amount of reversible lithium is preloaded into the SiO electrode. The ICE of the semi−cell Si−based pre−lithiation electrode is significantly increased to 90.7%, as shown in Figure 8e. In contrast to electrochemically driven lithiation (EDL), spontaneous etch−driven lithiation (SCDL) is fast and low cost, because once the anode is pre−lithiated, the battery assembly does not require a replenishment step [82]. Compared to EDL, SCDL results in more uneven pre−lithiation, because only the electrode region in direct contact with the lithium metal is pre−lithiated. Berhaut et al. found that additional Li^+^ were redistributed throughout the electrode by migrating to Si during the cycle, so that the preloading method could be used for an upgraded SCDL process [83]. They used SCDL of lithium metal on the anode surface of an a−Si/cFeSi_2_/graphite//LiNi_0.6_Mn_0.2_Co_0.2_O_2_ cell, with more than 1760 cycles at 100% discharge depth and less than 11% capacity loss at C/2.

### 3.2. Surface Engineering

**Figure 9:**
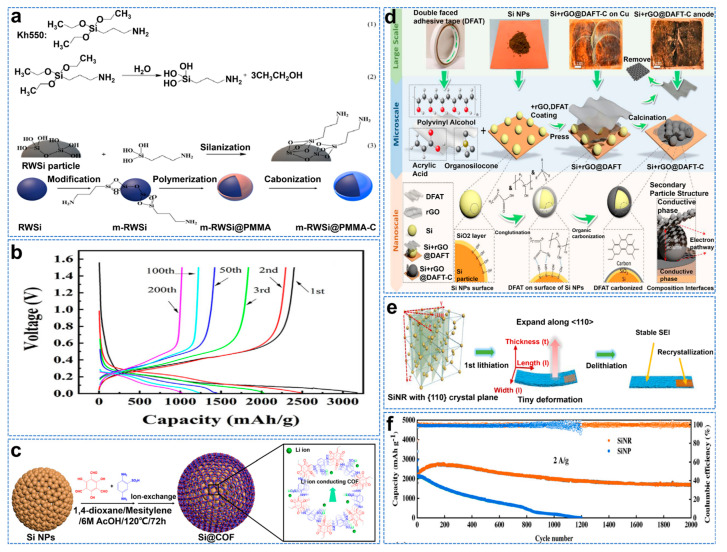
(**a**) Structure (1), hydrolysis reaction of kh550 (2) and schematic illustration of functionalized RWSi (3) and flow diagram of synthesis process for the m−RWSi@PMMAC composite, (**b**) Voltage profiles for different cycles at 0.2 A g^−1^ [87]. (**c**) Schematic illustration of preparation of Si@COF NPs [89]. (**d**) Schematic illustration of the process for Si+rGO@DFAT−C synthesis by a facile operation, depicting the process at scales of diverse lengths [91]. (**e**) Schematic diagram of ordinary Si anode material during the first lithiation/de−lithiation process, (**f**) Specific capacity of SiNP and SiNR at 2 A g^−1^ for 2000 cycles [93].

The continuous stability of SEI on the Si anode surface is a critical problem in practice. The reduction product of liquid electrolyte forms SEI passivation film due to the instability of liquid electrolyte at low potential. Although the Si nanostructure effectively alleviates volume expansion, many interfaces are generated [84]. In addition, nano Si tends to lead to uneven materials and forms dense areas on the particle surface, which are challenging to optimize. Park et al. used a pulsed laser to treat the dense surface area of an Si anode [85]. The laser surface treatment increased cycle life (1000 mAh g^−1^ at 200 cycles compared to the original 250 mAh g^−1^), and rate performance and Coulombic efficiency were significantly improved. The excellent electrochemical performance of Si@C anode is closely related to the surface area and crystal defects of the carbon coating. Therefore, it can be considered that a uniform small specific surface area and a high graphitization carbon layer positively affect electrical conductivity and electrochemical performance [86]. A dense carbon layer can alleviate volume expansion and isolate electrolytes to form stable SEI film. Shi et al. first used the silane coupling agent KH550 for surface engineering, functionalized RWSi particles through silanation reaction, and then modified and recycled Si powder (RWSi) as the core was wrapped in a protective layer, with PMMA as the carbon source (m−RWSi@PMMAC), as shown in Figure 9a [87]. The electrode had an initial capacity of 3176.2 mAh g^−1^ and an initial Coulombic efficiency (ICE) of 75.6% at 0.2 Ag^−1^, as shown in Figure 9b. Covalent organic frameworks (COFs) are two/three−dimensional polymers of atomic organization. The ordered pore structure in COFs provides a directional path for Li^+^ migration. In addition to good ionic conductivity, COFs also have excellent mechanical properties and low electronic conductivity [88]. Ai et al. successfully coated Si nanoparticles with a covalent organic framework (COF) through a two−step process, thus achieving excellent performance, as shown in Figure 9c [89]. The COF−coated Si has a high specific capacity of 1864 mAh g^−1^ at a high current density of 2 A g^−1^.

Double−interface engineering by constructing bonds between different components can positively impact the surface structure of anode materials [90]. Wang et al. encapsulated Si nanoparticles in a carbon–copper framework through a simple pyrolysis process, as shown in Figure 9d [91]. Due to Cu−O−C, Si−O−C, and Si−C chemical bonds, Si−hybrid reduced GO (rGO) and double−sided−tape−derived carbon composites (Si+rGO@DFAT−C) have high structural integrity and immune stratification. As such, they exhibit excellent capability (1536 mAh g^−1^ at 0.1 A g^−1^) and rate capability (1126 mAh g^−1^ at 2 A g^−1^). The lithium dynamic of crystalline Si is anisotropic, which is accompanied by the change of phase transition and mechanical properties and is closely related to the rate and direction of lithium, with <111> reported to be the slowest and <110> to be the fastest [92]. Zhang et al. used the electrochemical micromachining method to synthesize a new Si nanoribbon (SiNR) with a (110) crystal face as the anode material of a lithium−ion battery, as shown in Figure 9e [93]. After 2000 cycles at 2A g^−1^, the specific capacity is 1721.3 mAh g^−1^(~80% capacity retention) and the initial Coulombic efficiency (ICE) is 83%, as shown in Figure 9f.

### 3.3. Binders

**Figure 10:**
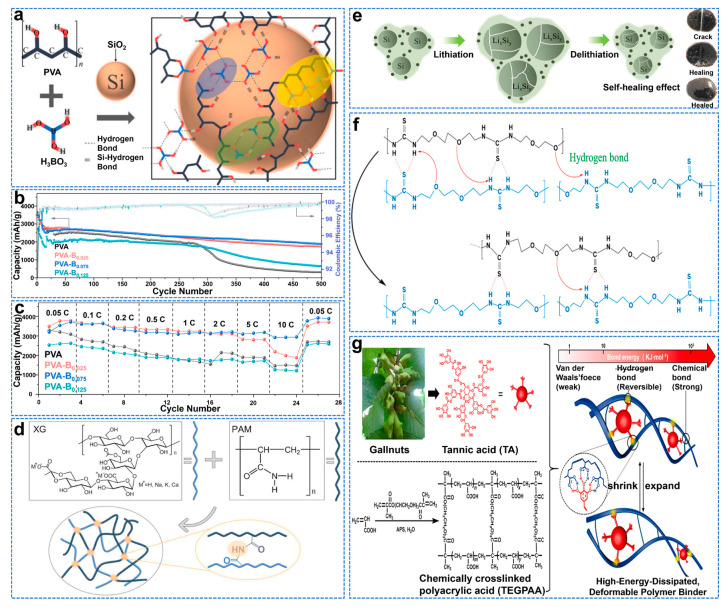
(**a**) Schematic of the cross−linking mechanism for PVA−B complexes and the interaction with the −OH on Si surface, (**b**) Long−term cycling performances, (**c**) Rate capability for the PVA−B binder at different BA molar fractions prepared at 40 °C for 10h [97]. (**d**) Schematic diagram of the cross−linked reaction between XG and PAM [101]. (**e**) Schematic representations of lithiation/de−lithiation of Si particles using the SHPET binder, (**f**) The self−healing mechanism: the slip motion of hydrogen bonds on SHPET backbones enables the patching function [105]. (**g**) The synthesis of highly−energy−dissipated and deformable polymer binder (TEGPAA−TA) for Si anode [106].

The binder plays a vital role in the connection between electrodes and materials. However, the traditional polyvinylidene fluoride (PVDF) binder has low tensile strength and weak Van Der Waals binding force, so it cannot provide enough adhesion to resist the severe volume change of Si after a long cycle [94]. The PVDF solvent NMP (N−methyl−2−pyrrolidone) is volatile, explosive, and will cause environmental problems. At the same time, polyvinyl alcohol (PVA) has high water solubility and high thermal stability (melting point 230–240 °C). Its main carbon chain contains abundant hydroxyl groups, making it easy to interact with other functional groups such as −OH and −COOH [95]. On the other hand, the B element can form a stable SEI film by forming a large O−B−O on the Si surface [96]. Cao et al. designed a three−dimensional (3D) adhesive network using boric acid (BA) cross−linked polyvinyl alcohol (PVA) as the skeleton, as shown in Figure 10a [97]. Due to the dehydration of the Si−OH group, the B−OH bond is connected between the binder and the Si surface. A high ICE (92.76%) was obtained, and the capacity of the Si anode remained at 1883.7 mAh g^−1^ after 500 cycles, as shown in Figure 10b, much higher than that of the pure PVA Si anode (~317.9 mAh g^−1^). As shown in Figure 10c, at 10 C rate (corresponding to 42 A g^−1^), the Si anode using the PVA−B_0.075_ binder delivers the highest discharge capacity of 2920.73 mAh g^−1,^ significantly higher than the 1481.33 mAh g^−1^ obtained with pure PVA binder.

Polyacrylic acid (PAA) can interact with Si particles to provide sufficient physical support to improve the mechanical stability of the electrode [98]. Wang et al. used hydroxyl (−OH) in borax to react with the carboxyl group (−COOH) of polyacrylic acid (PAA) to generate three−dimensional polymers [99]. After 50 cycles, its capacity is about 2470 mAh g^−1^, and its capacity retention rate is 91.2% after over 500 cycles with capacity of 1000 mAh g^−1^. Xanthan gum, a natural polysaccharide deriving from the fermentation process of Xanthomonas campestris, contains a linear cellulosic backbone of (1,4)−β−D−Glucose units with trisaccharide side chains [100]. However, linear polymer chains tend to move along the surface of Si nanoparticles, leading to the deterioration of electrode performance. Zhang et al. developed a water−soluble polymer binder with a three−dimensional network by in situ thermal cross−linking of xanthan gum (XG) and polyacrylamide (PAM), as shown in Figure 10d [101]. The cross−linking structure of C−XG−PAM gel binder containing hydroxyl and amide groups significantly improves the adhesion to Si and copper current collectors. Therefore, C−XG−PAM binder is used in a SiNP electrode (1104 mAh g^−1^ after 1000 cycles).

Natural adhesives have the advantages of being sustainable, low cost, of high molecular weight, and with great bonding groups such as carboxyl, hydroxyl, and amino, making them ideal in applications for Si electrodes. Wang et al. modified the natural vegetable gum, guar gum (GG), by a carboxymethylation method to obtain carboxymethyl guar gum (CMGG) with quick water solubility, good thermal stability, high viscosity, and strong adhesion [102]. The Si@CMGG electrode maintains a high reversible capacity of 1865 mAh g^−1^ after 200 cycles and has a capacity decay rate of 0.12% per cycle. The self−healing adhesive can continuously repair the cracks caused by the volume expansion of the Si anode. However, its mechanical and repair properties cannot work together, so the adhesive is easy to deform [103]. Hydrogen bonding pairs can achieve the repair function of SHPET polymers, while SHPET polymer crosslinked thiourea units can achieve good rigidity to balance the softness of self−healing polymers containing hydrogen bonds [104]. Chen et al. synthesized a novel self−healing polyether thiourea (SHPET) polymer with balanced rigidity and softness, as shown in Figure 10e,f [105]. It provides a high discharge capacity of 3744 mAh g^−1^ at a current density of 420 mA g^−1^. At a high current rate of 4200 mA g^−1^, the capacity retention rate is 85.6% after 250 cycles, and the cycle life is stable. The “static” (chemical cross−linking) and “dynamic” (physical cross−linking) synergies can dissociate non−covalent hydrogen bonds, extensively promoting the dispersion and release of adverse stress, while the permanent chemical bond network enables the deformable network to maintain the electrode structure. Hu et al. constructed a three−dimensional network by polymerizing acrylic acid and trimethylene glycol dimethacrylate (chemical crosslinking agent) [106]. They then introduced high−branched tannic acid (TA) as the second physical crosslinking agent through hydrogen bond integration in order to obtain a better silica−based anode, as shown in Figure 10g.

### 3.4. Electrolyte and Electrolyte Additives

The electrolyte is critical for regulating the SEI layer properties and the electrochemical performance of LIBs. The LiPF_6_ salt currently used will hydrolyze at high temperatures or in the presence of water to produce HF, which will not only erode the silicon−based electrode and accelerate cell failure, but also make it difficult to maintain the interfacial stability of the silicon−based electrode. There are usually two solutions: one is to change the electrolyte, and the other is to add additives to the original electrolyte [107].

LiFSI has better thermal and chemical stability compared to LiPF_6_ and can form LiF−rich SEI layers to prevent the formation of HF. Liu et al. designed a fluorinated ethylene carbonate (FEC) double salt (LiFSI−LiPF_6_) ether−based localized high concentration electrolyte (D−LHCE−F) [108]. The results show that the LiFSI salt prevents the formation of corrosive HF, while the addition of FEC improves the interfacial stability by promoting the formation of protective LiF−rich SEI and increasing the flexibility of the interface. The SiO_x_/C electrode using the unique D−LHCE−F maintains up to 78.5% capacity retention after 500 cycles at 0.5 °C, far exceeding that of the control electrolyte (3.4% capacity retention). The use of fluorinated carbonates as electrolyte solvents may be a new strategy to improve the safety of lithium batteries. On the other hand, compared with common dilute electrolytes, concentrated electrolytes have attracted much attention in recent years because they contain fewer free−solvent molecules and are more reactive to form a strong passivation film and mitigate the degradation of electrodes and electrolytes. Zeng et al. prepared a mixed−fluorinated−carbonate−solvent composite electrolyte with FEC and TFEC using 3.5 M LiFSI as salt [109]. The results show that the electrolyte not only improves the cycling stability of silicon nanoparticle (SiNPs) anodes, but also solves the safety hazards caused by high temperatures.

The design of electrolyte additives by optimizing the electrolyte components has been considered in recent years one of the most economical, feasible and scalable approaches to improve the electrochemical performance of silicon−based anodes [110]. Sulfur−containing molecules are considered one of the most promising electrolyte additives due to their preference for electrochemical reduction and the fact that sulfur−based compounds contribute to the formation of stable and favorable SEI. Liu et al. effectively suppressed the parasitic side reactions at the electrode–electrolyte interface by using allyl phenyl sulfone (APS) as an electrolyte additive [111]. Meanwhile, the stronger binding energy between APS and H_2_O/HF/PF_5_ helped to further enhance the interfacial stability by stabilizing LiPF_6_. The cycling stability of the SiO_x_/C electrode was significantly improved after the addition of 0.5 wt% APS, with a capacity retention of 777 mAh g^−1^ (79.3% capacity retention) after 200 cycles at 0.5 C and 30 °C, which far exceeded that of the baseline electrolyte (57.8% capacity retention). Ghaur et al. investigated the synergistic effect between FEC and hexafluorocyclotriphosphazene−derivatives (HFPN), which effectively stabilized the formation of SEI, led to an overall lower cell impedance and enhanced the electrochemical performance of SiO_x_ anodes [112]. The electrolyte mixture with the dual additive approach was able to stabilize the electrolyte at high temperature (60 °C). The use of HFPN derivatives as additives inhibits the decomposition of ethylene carbonate and ethyl methyl carbonate, transesterification and oligomerization product reduction.

### 3.5. Collectors

In conventional LIBs, copper foil is usually used as the current collector for the anode, which is a very heavy and rather expensive component. With the addition of other inactive components in the electrode assembly, it reduces the total energy density and increases the manufacturing cost of LIBs [113].

Melamine formaldehyde foam (MF) is a commercial polymer foam widely used because of its low cost, low density, high porosity and environmental friendliness. Carbon foam with three−dimensional (3D) network structure can be easily obtained by carbonizing MF in an inert atmosphere, but its practical application is hindered by its poor electrical conductivity. Liu et al. obtained a self−supporting silicon electrode for lithium−ion batteries by modifying carbonized MF with a thin titanium layer that can be used as a cheap and lightweight current collector, and depositing a silicon film on its surface [114]. The annealing treatment was able to further strengthen the Ti/Si interface, which greatly suppressed the electrode chalking caused by the great volume change of silicon during cycling. The self−supported silicon electrode showed a high charge specific capacity of 1296 mAh g^−1^ and excellent cycling performance of up to 1000 cycles at a current density of 2.0 A g^−1^.

To minimize the electrical contact losses between the silicon active material and the current collector, several studies have been conducted to optimize the structure of the current collector, and the cycling performance of the roughened silicon anode has been improved. This improvement is due to better adhesion between the active material and the substrate [115]. Chen et al. studied the effect of collector surface roughness on the performance of thin−film silicon anodes [116]. Lower Coulombic efficiency of pristine Cu samples compared to FeCl_3_ etching was mainly due to the loss of contact of the active material due to cracks caused by larger volume changes. On the other hand, FeCl_3_ etched the surface of the test sample and the rougher surface enhanced the adhesion of the film to the collector interface, which maintained the structural stability in the presence of Si volume expansion.

For commercial electrodes, the lithium–anode interface can move up to tens of microns during cycling. After continuous cycling and precipitation, the relative volume of the lithium anode changes infinitely, blocking ion transport and leading to excessive gap formation between the porous electrode and the dead lithium, resulting in capacity loss [117]. As a result, all these factors lead to poor physical contact at the interface, high interfacial resistance, uneven current distribution, and large cell polarization during continuous cycling. To solve this problem, some groups have used elastic elements with high external pressure to eliminate the Li_2_CO_3_ layer. Liang et al. proposed a new strategy to prepare elastic deformable current collectors with highly conductive Cu surfaces using a polydopamine−assisted chemical metallization process [118]. The deformable current collectors can not only act as artificial springs to stabilize the interfacial stresses, but also improve the conductivity of the anode without using any binder or conductive agent. The surface resistance is as low as 0.06 Ω/sq.

## 4. Advanced Characterization Techniques and Mechanisms

The volume and surface properties of Si−based electrodes are constantly changing during lithiation/de−lithiation. In order to better solve the problem of the Si anode during cycling, it seems imperative to combine various characterization techniques to clarify their lithiation/de−lithiation mechanisms.

### 4.1. Morphological Changes and Lithiation Mechanism of a Pure Si Anode

**Figure 11:**
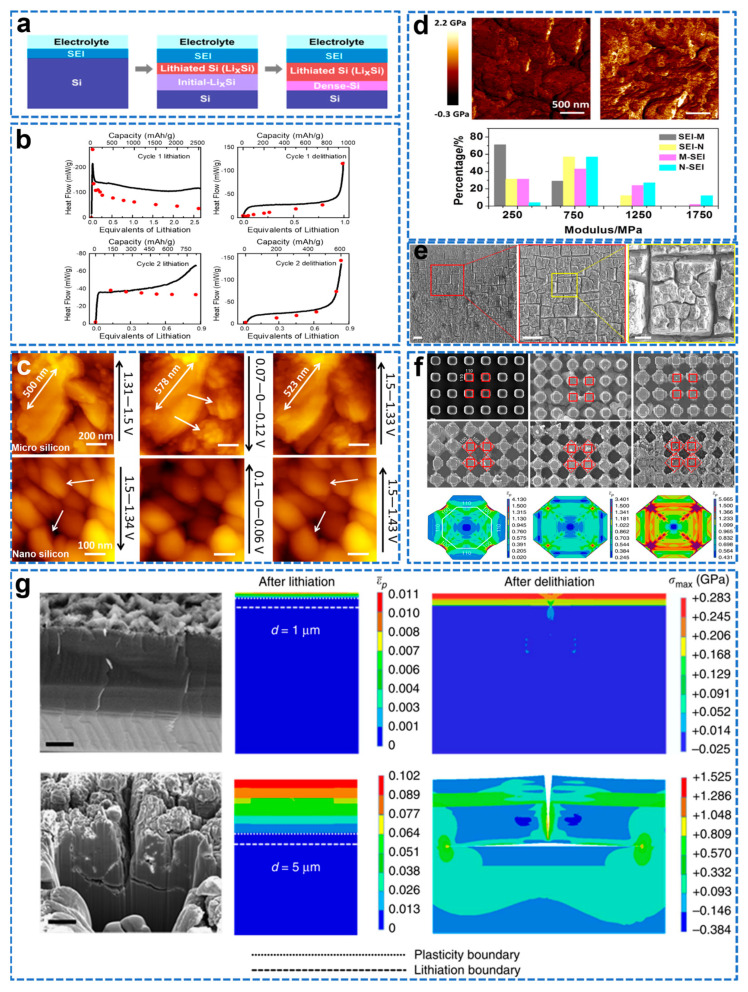
(**a**) Sketch of the three−step lithiation process [119]. (**b**) Comparison of IMC−measured total heat flow (black) and the sum of polarization and entropic heat flow (red) [121]. (**c**) In situ AFM images of Micro−Si and Nano−Si electrode cycled at a scanning rate of 0.6 mV s^−1^ between 1.5 and 0.0 V in the 1 M LiPF_6_/EC/DMC, (**d**) Modulus images of SEI−M, SEI−N and Young’s modulus distributions of SEI−M, SEI−N, M−SEI and N−SEI [123]. (**e**) Surface changes after 30 cycles, (**f**) Scanning electron microscope (SEM) images of square micropillars fabricated on a p−type Si(100) wafer obtained at various lithiation stages and finite element results, (**g**) Cross−sectional focused ion beam (FIB)−scanning electron microscope (SEM) images (left column) and corresponding finite element method (FEM) results (middle and right columns) of a Si(100) electrode obtained after 3 and 50 lithiation/de−lithiation cycles [124].

In situ surface sensitive X−ray reflectivity (XRR) enables real−time observation of the lithiation reaction at the interface during lithiation at sub−nanometer (sub−nm) resolution. Cao et al. studied the lithiation process of a single crystal Si <100> electrode in the first discharge cycle by in situ XRR for the first time and found that the thickness of the Li_x_Si layer is proportional to the charge, and proposed a three−stage lithiation model, as shown in Figure 11a [119]. First, SEI begins to form between 0.8–0.6 V. Second is lithiation of native oxides and initial diffusion of Li to Si. Finally, there is lithiation of bulk Si to a phase close to Li_15_Si_4_. Isothermal microcalorimetry (IMC) is a powerful tool to study battery reactions by detecting the heat flow generated during charging and discharging [120]. Combining IMC data with electrochemical measurements provides insight into the failure mechanism of Si electrodes in the first few cycles of extremely low ICE. Housel et al. performed a calorimetric study of parasitic heat and polarization during lithiation/de−lithiation of crystalline Si by nondestructive in situ isothermal microcalorimetry (IMC) to determine the onset of parasitic reactions [121]. Combined with in situ X−ray diffraction (XRD), it showed that the amorphization transition heat at the first lithiation had a significant contribution to the entropic heat flow term. As shown in Figure 11b, the parasitic heat starts at the first lithiation (25 mAh g^−1^), and the contribution of the parasitic heat flow to the total heat flow increases as the first lithiation progresses. During the first lithiation, parasitic heat contributed about 70% of the total heat flow at 2500 mAh g^−1^. The collection and analysis of heat flow data by IMC can be adapted to track parasitic reactions occurring in the battery. In situ AFM is one of the most powerful tools to directly study electrode interface reactions by applying a function of electrochemical potential/electrolyte composition/additives to monitor the evolution of surface topography in real time with nanoscale resolution [122]. Huang et al. used in situ electrochemical atomic force microscopy (EC−AFM) with different working modes to directly observe the surface morphology of nano/micro Si, as shown in Figure 11c [123]. The nano/micro−Si anode remained intact at the initial stage of lithiation. With the lithiation process, the larger volume expansion of micro Si leads to cracks on its surface, so that the SEI grows continuously, and the SEI formed on the micro Si electrode is thicker. In contrast, nano−Si is more stable. As shown in Figure 11d, the SEI film on the surface of the micro Si electrode was analyzed by the Young’s modulus, which was softer than the SEI on the surface of the nano Si electrode. Ex situ XPS analysis shows that the SEI on the nano Si surface is mainly composed of LiF and carbonates, and its higher specific surface area contains more SiO_x_, which is easily formed SiF_x_ with F^−^, reducing the organic species and forming a harder SEI. AFM combined with ex situ XPS is suitable for studying the evolution of morphology, structure and mechanical properties of different Si electrode surface structures. Shi et al. employed CV testing combined with structural characterization, mechanics, and finite element analysis (FEM) to reveal the fundamental fracture mechanism of single−crystal Si electrodes during electrochemical cycling [124]. As shown in Figure 11e, surface cracks appear in two alternate directions after 30 cycles, forming isolated quadrilateral shapes, and after 50 cycles, forming quadrilateral voids that eventually lead to delamination of the electrode surface. As shown in Figure 11f, lithiation will preferentially occur in the <110> direction, and significantly less in the <100> and <111> directions. As the degree of lithiation increases, expansion is preferentially from the <110> direction, while the expansion of the rounded corners decreases significantly, allowing adjacent micropillars to merge. Due to the faster lithiation rate in the <110> direction, the <110> is subjected to higher compressive forces, which results in high shear stress and plastic direction along the <100> direction. In order to verify the above conclusions, the temperature field was used to simulate the electrochemical process of lithium ions in the Si electrode, and the intrusion rate of lithium in the <110> direction was 6.4 times that in the <100> direction. As shown in Figure 11g, the cross−sectional SEM image of the surface crack after 50 cycles, the crack started at the electrode surface and propagated in the depth direction as the lithiation progressed, and after reaching a depth of 5–8 mm, deflected to the lateral direction; from the perspective of energy and mechanics, it is found that the crack deflects along the a−Si/c−Si interface, which is the weakest direction, which is more favorable in energy and mechanics. These results promote the commercial application of Si anodes.

**Figure 12:**
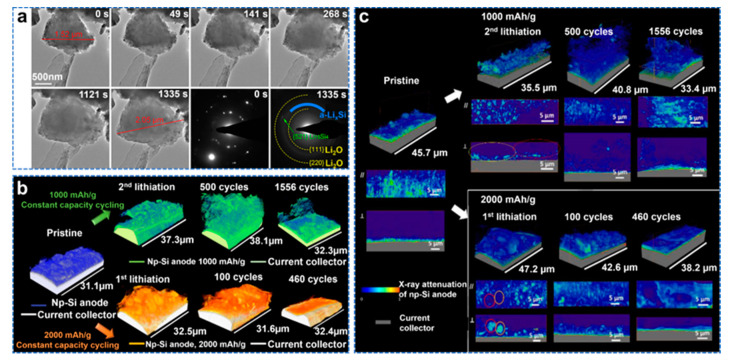
(**a**) In situ TEM observation of the lithiation process of a porous Si particle [125]. (**b**) X−ray nanotomography reconstruction with volume rendering shows the morphological evolution of the np−Si anode, (**c**) Quantitative material density 3D maps and pseudo cross sections from X−ray nanotomography showing electrode cycled in constant capacity mode [127].

Porous Si material has become a promising anode material due to its stable cycling performance. In situ transmission electron microscopy (TEM) can provide important information on the structural changes and lithiation kinetics of Si during lithiation/de−lithiation. Shen et al. observed the lithiation behavior of porous Si nanoparticles and porous Si nanowires using in situ and ex situ TEM [125]. As shown in Figure 12a, a diameter of porous Si nanoparticles up to 1.52 µm was selected. At the beginning of lithiation, the particles start to expand in volume from the lower right corner, and then to the upper left corner. Due to the large specific surface area of porous Si, overall, it lags behind the propagation of lithium in the particles. Lithium ions flow in an end−to−end fashion in porous Si nanoparticles, unlike Si particles during lithiation from the surface to the center. After lithiation for 1335 s, the particle diameter increased to 2.05 µm, and no cracks were observed, indicating that the critical fracture diameter of porous Si particles was as high as 1.52 µm, which was larger than 150 nm for crystalline Si nanoparticles and 870 nm for amorphous Si nanoparticles many. TXM was combined with other techniques for complementary analysis [126]. Zhao et al. prepared nanoporous Si (np−Si) by liquid metal dealloying (LMD) and used synchrotron radiation X−ray nanotomography to study the morphological transformation and failure mechanism of porous Si [127]. Morphological changes are shown as 3D X−ray nanotomography results of electrochemical cycling of porous Si, as shown in Figure 12b. Under the 1000 and 2000 mAh g^−1^ cycling conditions, the porous Si particles were not uniformly lithiated in the early stage of lithiation, which resulted in uneven distribution of porous Si after lithiation and thickness variation at the macroscopic level. This can lead to uneven stress distribution and unstable mechanical properties. Si pulverization was also observed in the porous Si structure under both cycling conditions. However, this crushing did not cause the battery to fail. Si powdering was observed. The interaction between different lithiation ligaments during the volume expansion process and stress changes during charging and discharging all lead to porous pulverization, but porous Si pulverization does not lead to significant capacity loss. As in Figure 12c, showing non−uniform X−ray attenuation, during cycling, partially lithiated Si can be observed in the electrode, suggesting that the less reactive porous Si would be restricted from lithiation. This can be explained by the lithium−ion diffusion process, which occurs more slowly away from the electrode/electrolyte interface. Agglomeration was also observed. Under the 1 and 2 A g^−1^ cycling conditions, the porous Si results in different failure rates due to different degrees of agglomeration. At 2 A g^−1^, the agglomeration leads to uneven stress distribution, which hinders the activation of active materials and the diffusion of lithium ions. This results in a gradual delamination of the active material from the electrode. Eventually, when fully delaminated, the structure fails.

### 4.2. Structural Controlling and Lithiation/De−Lithiation Mechanism of Si/C Composites

**Figure 13:**
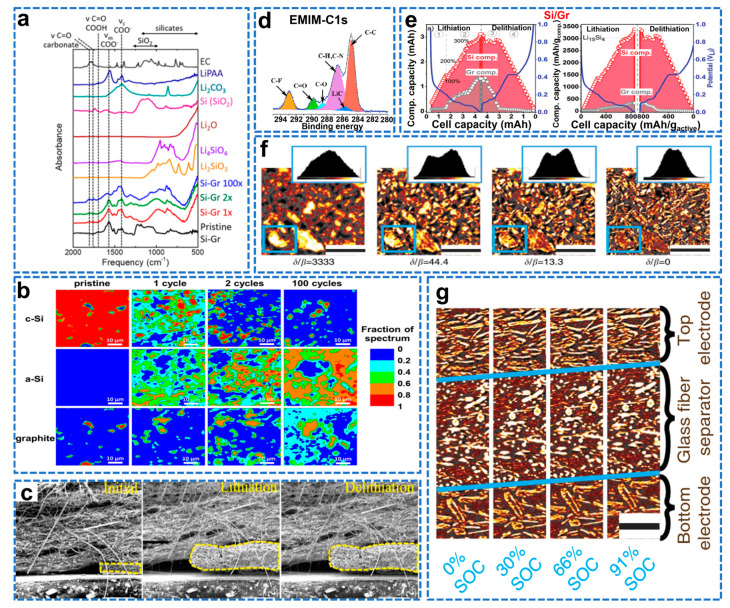
(**a**) FTIR spectra of the pristine Si–Gr anode and the Si–Gr anodes after 1 cycle, after 2 cycles, and after 100 cycles, (**b**) RAMAN maps of Si–Gr anodes taken before cycling (pristine), after 1 formation cycle, after 2 formation cycles, and after the full cycling protocol (100 cycles) [129]. (**c**) Morphological evolution of Si/C electrodes at 60 °C and in situ SEM images for EMI, (**d**) C1s high−solution XPS spectra of surface components formed on Si/C electrodes after 20 cycles with corresponding ionic liquids in coin cell at 60 °C [130]. (**e**) Capacity and specific capacity of the Gr and Si components in a Si–Gr electrode during lithiation and de−lithiation [131]. (**f**) Comparison of a slice in the bottom electrode, where the phase has been retrieved with different ratios. Insets show the respective color−scale histograms, (**g**) Vertical cuts through the sample at different states of charge [132].

Si/graphite composites are considered to be the most viable alternative materials for next−generation anodes due to their excellent electrochemical properties. X−ray technology is widely used to probe the morphological transformation during electrochemical cycling of various electrode materials. Finegan et al. combined high−speed X−ray diffraction (XRD) and XRD computed tomography (XRD−CT) for the first time to reveal the charge dynamics between Si and graphite at 1 µm image resolution and 0.01s temporal resolution, observing inhomogeneity of lithiation of Si and graphite particles [128]. The crystalline Si core in the lithiation shell exists in the discharged state. The presence of the crystalline Si core indicates that most of the battery capacity remains, thereby reducing the effective energy density of the electrode. Since stress is generated after lithiation of Si, compressive stress is applied to the crystal nucleus at the interface between the lithiation phase and the non−lithiation phase. The lithiated Si shell and crystalline Si core were segmented in XRD−CT images, and their respective diffraction curves were plotted; the diffraction profile of the lithiation shell structure was significantly broadened and not completely amorphous as reported in the literature, while showing the characteristics of crystalline Si and metastable lithium silicide. XRD−CT scanning was performed after the electrode was de−lithiated. The phase diagram shows that after the de−lithiation process, the crystalline Si phase still exists in the electrode. However, compared with the core–shell structure observed during lithiation, both regions are shrunk. A combination of high−speed X−ray diffraction (XRD) and XRD−computed tomography (XRD−CT) can elucidate the relationship between the inhomogeneity of lithiation rates and failure mechanisms in electrodes and individual particles. Vibrational spectroscopy can identify different mechanisms of electrochemical performance loss, whereas most studies have only examined half−cells of Si−graphite composites. Ruther et al. combined FTIR and RAMAN to study the energy loss mechanism of LiNi_0.5_Mn_0.3_Co_0.2_O_2_ (NMC532) full cells [129]. FTIR analysis showed that the chemical composition of the SEI was significantly different after long−term cycling of the full cell. The main reason for the capacity fading of the full cell with a Si–Gr anode is the loss of cyclable lithium. As shown in Figure 13a, FTIR identified several side reactions that consume cyclable lithium. As shown in Figure 13b, RAMAN micro−spectroscopy combined with cluster and basis analysis assessed the heterogeneity of electrode composition and electrochemical reactivity. After cycling, some inactive crystalline Si remained in the anode region and did not participate in the alloying reaction with lithium.

The working temperature of the battery is about 20–60 °C; however, most of the mechanism studies were carried out at room temperature, which inevitably deviates from the practical application. Wu et al. used in situ environmental scanning electron microscopy (ESEM) to study the morphological evolution of the electrolyte−based Si–graphite composite electrode in the range of 20–60 °C [130]. As shown in Figure 13c, at 60 °C, the volume expansion rate of the EMI electrode is 152%, far exceeding its theoretical value (67.8%), indicating that the dramatic volume expansion is not only due to the conventional lithiation of Si. Due to the amorphization of crystalline Si during lithiation, it is difficult for conventional detection methods to understand its lithiation mechanism. To better distinguish the volume expansion extent of Si/C electrodes when using EMI and PYR as electrolyte at different temperatures, difference in volume expansion ratio between EMI cycled electrodes is investigated. As shown in Figure 13d, combining transmission electron microscopy (TEM) and X−ray photoelectron spectroscopy to analyze its microstructure and surface reaction products, it was found that the huge volume expansion was caused by the side reaction between the decomposition of graphite and electrolyte to generate C−H and C−F bonded organic molecules, causing a dramatic volume expansion, hindering further lithiation of Si.

Yao et al. used energy−dispersive X−ray diffraction to quantify the lithiation and de−lithiation of Si and Gr particles in a 15 wt% Si composite electrode [131]. By combining lithiation/de−lithiation knowledge with battery capacity information, the dominance of Si and graphite in the lithiation process can be inferred. As shown in Figure 13e, during the initial lithiation process (1.0–0.20 V), Si is alloyed with lithium, and the lithiation rate of Si is much higher than that of graphite (0.96/0.04). When the electrode potential is 0.01–0.2V, the lithiation rate of Si is slightly higher than that of graphite (0.58/0.42). During the initial de−lithiation process (0.01–0.22V), almost all Li ions are extracted from Gr. When the potential is 0.22–1.0 V, the Li–Si lithiation rate is much higher than that of graphite (0.97/0.03). The de−lithiation of Si–Gr electrodes starts from Gr first and then from Si. These trends can be used to rationally choose the electrochemical cycle window and limit the volume expansion of Si particles, thereby optimizing the commercial application of Si anode Li−ion batteries. Synchrotron X−ray tomography (XTM) non−invasively images electrode volume expansion and attenuation refinement changes with high spatial and temporal resolution. Pietsch et al. investigated weak decay during lithiation/de−lithiation of graphite electrodes by in situ tomography (XTM) and combined digital volume correlation to capture electrode dynamics [132]. As shown in Figure 13f, describing the Operando XTM measurement setup, higher ratios of δ/β can improve the grayscale contrast between particles and background, but also reduce the effective spatial resolution. As shown in Figure 13g, the transport of lithium through the pore space is also restricted, resulting in a lithium−ion front and a large SOC gradient, and the expansion in the electrode is strongly anisotropic, which originates from the directional expansion of the graphite. In conclusion, XTM can be used to study the lithiation mechanism and structural changes of Si–graphite composite anodes during lithiation/de−lithiation.

### 4.3. Formation Mechanism of SEI on Si−Based Anodes

A stable SEI can suppress electron transfer and allow lithium ions to diffuse for reaction. However, the volume expansion of Si during lithiation is huge, which makes the SEI unstable and continuous, and consumes the electrolyte continuously, thus affecting the electrochemical performance. The Si reacts with the electrolyte to form insoluble products, while the SEI passivates the electrode and suppresses side reactions to prevent lithium depletion, allowing the electrode to cycle stably. Therefore, the stability of the SEI film determines the cycling performance of Li−ion batteries. Since its thickness is 10–50 nm [133], it is difficult to extract a sufficient amount of SEI for subsequent compositional analysis. At the same time, the weak SEI signal is difficult to distinguish from the material. In addition, lithium, fluorine, and organic compounds in SEI are highly reactive and chemically volatile, so it is always challenging to characterize SEI in the real, invariant state.

**Figure 14:**
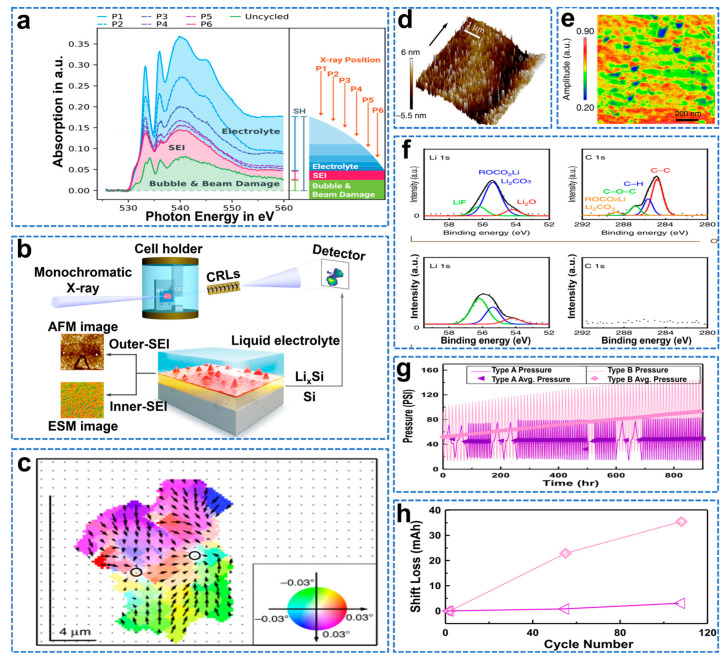
(**a**) Oxygen K−edge transmission XAS spectra of cycled cells at six different sample positions P1 to P6 alongside one spectrum of an uncycled cell, (**b**) FFDXM working principle and operando electrochemical cell configuration, (**c**) Results of 3D RSM (direction and magnitude of lattice tilt are indicated by false colors and arrows), (**d**) Topographical map during the resting period, (**e**) ESM amplitude map, (**f**) Li 1s and C 1s sputter−etched XPS spectra of Si electrodes after the 6th cycle [135]. (**g**) Pressure versus time and average discharge pressure versus time for type A NMC/Graphite and type B LCO/Graphite, (**h**) Displacement loss versus cycle number obtained from the dV/dQ fit [137].

Using a soft X−ray transparent electrolyte layer, Schellenberger et al. identified acetate (based on lithium acetate and lithium trifluoroacetate), lithium ethylene carbonate (based on LEMC and LEDC), pyruvate based on the spectral XAS fingerprint of the oxygen K−edge (based on lithium acetylacetonate) and LiOH as the most likely solid species present in the SEI on the Si anode, as shown in Figure 14a [134]. At the fluorine K−edge, they determined that LiF is the dominant fluorine−containing species in the SEI. In addition to this, they suspect that the acetate species may be fluorinated and thus correlate with the observed trifluoroacetic acid information. Furthermore, unlike other literature, evidence of aldehyde species was found and attributed to the possible presence of liquid inclusions in the porous SEI morphology. Chen et al. used full−field diffractive X−ray microscopy (FFDXM) to observe SEI defect formation in real time and correlate it with the lithiation process, as shown in Figure 14b [135]. Furthermore, atomic force microscopy (AFM), electrochemical strain microscopy (ESM), and sputter−etching X−ray photoelectron spectroscopy (XPS) were combined to investigate the link between the lithiation process and SEI inhomogeneity. The most severe part of the deformation is mainly caused by the lithiation/de−lithiation early defects (density <100 ppm, small deformation amplitude), and suppressing the early defects can improve the cycling stability of the Si anode, thereby promoting its commercial development. As shown in Figure 14c, the 3D RSM shows the processing defect region with the lattice tilted inward, indicating that the degree of lithiation in this part is lower than that in the surrounding region, and this non−uniform lithiation may be caused by the non−uniform SEI thickness. During the subsequent open circuit, a complete topographic image was taken, as shown in Figure 14d. The results show that the surface is covered by a non−uniform SEI layer with an RMS roughness of 2.4 nm. As shown in Figure 14e, ex situ ESM results show that the Li−ion mobility of the SEI layer varies greatly, resulting in a huge difference in lithium−ion conductivity. The SEI formed on Si has a double−layer structure consisting of a softer organic outer SEI layer and a harder inorganic inner SEI layer. The outer SEI layer is mainly composed of Li_2_CO_3_ and ROCO_2_Li, as shown in Figure 14f, and its electrical conductivity is higher than that of LiF (the main component of the inner SEI), resulting in a higher lithium−ion mobility in the outer SEI layer than in the inner SEI layer [136]. This indicates that the inner SEI layer is the main factor limiting lithium−ion transport. Li et al. performed pressure measurements on coin cells using non−destructive in situ battery pack pressure measurements to reveal a correlation between irreversible volume expansion and capacity loss due to continuous unstable growth of the SEI [137]. As shown in Figure 14g, during cycling, the irreversible pressure evolution caused by irreversible volume expansion begins to develop. As shown in Figure 14h, the mechanism of lithium loss is analyzed, and the main source of capacity loss in the battery is the anode SEI growth. It is suggested that the anode SEI growth is responsible for the irreversible volume expansion detected by the operand pressure measurement.

**Figure 15:**
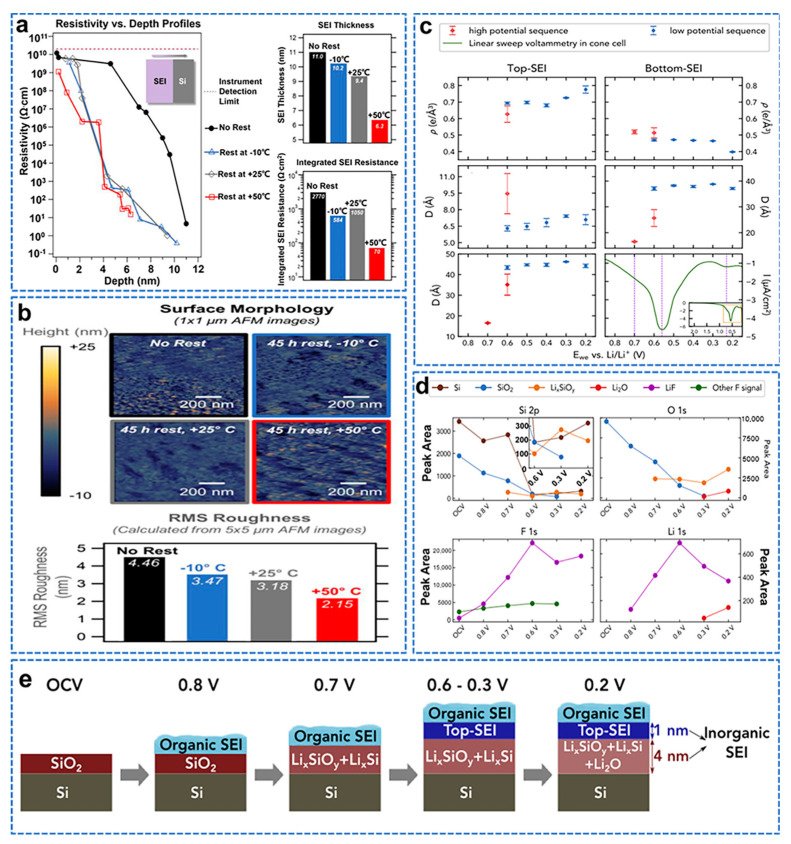
(**a**) Comparison of SSRM 3−D resistivity vs. depth profiles, SEI thickness and integrated SEI resistance obtained from resistivity vs. depth profiles for SEI with varied resting protocols, (**b**) AFM images at 1 × 1 µm showing surface morphology of SEI with 50 cycles of SEI formation with varied resting protocols and RMS roughness from the same surfaces, calculated from larger 5 × 5 µm AFM images [139]. (**c**) Best−fit results of XRR datasets of high−potential (red markers) and low−potential (blue markers) sequence experiments, (**d**) Voltage−dependent XPS fit−derived peak areas corresponding to Si 2p, O 1s, F 1s, and Li 1s spectral ranges, (**e**) Schematic illustration of the proposed potential−dependent SEI growth mechanism on native oxide−terminated Si anodes [140].

Understanding the response of the SEI to temperature is critical, as any temperature−induced instability will limit the passivation of the electrode, prevent further reactions with the electrolyte, and may have a large impact on Li−ion battery failure [138]. Stetson et al. combined electrochemical tests, three−dimensional resistivity, depth profiles, and atomic force microscopy to show that SEI dissolves or dissociates at a faster rate at higher temperatures [139]. The SSRM 3−D resistivity versus depth profile shows that the resistive component is reduced throughout the SEI, the SEI thickness is reduced during rest, and the thickness loss is greater at higher rest temperatures, as shown in Figure 15a. AMF also confirmed that the quiescence of the SEI resulted in a decrease in surface roughness, as shown in Figure 15b. Cao et al. combined in situ synchrotron radiation X−ray reflectivity, linear sweep voltammetry, ex situ X−ray photoelectron spectroscopy, and first−principles calculations [140]. The elucidated the formation process and structure of the solid electrolyte interface (SEI) layer on the carbonate−based electrolyte (LP30) natural oxide Si wafer anode. The SEI layer thickness and electron density in the high−potential and low−potential sequential XRR experiments are summarized, as shown in Figure 15c. The top SEI starts to grow at 0.6 V and increases in thickness and density as the potential decreases. The bottom SEI appears at 0.7 V, grows all the way to 0.6 V, and then maintains a constant thickness, but the density drops to 0.3 V towards lower potentials. At 0.2 V, the density of the bottom SEI layer decreases significantly, which is consistent with the formation of Li_2_O; the final thickness of the top SEI layer is about 7 Å, and that of the bottom SEI layer is about 38 Å. Combined with the XPS spectrum, it is shown in Figure 15d. The initial formation of Li_x_SiO_y_ starts at about 0.7 V. The main contribution to electrolyte decomposition is concentrated at 0.6 V and leads to the formation of LiF and Nd on top of Li_x_SiO_y_. Li_2_O is formed between 0.3 and 0.2 V. These observations are consistent with our XRR results, which indicate that a low−density bottom SEI layer is formed at 0.7 V, a high−density top SEI layer is formed at 0.6 V, and the bottom SEI layer is changed around 0.2 V. By combining in situ XRR, LSV, ex situ XPS, and FPC, they present a detailed picture of the SEI formation process on Si anodes, as shown in Figure 15e.

### 4.4. Optimal Surface Oxide Layers of Si−Based Anodes

The surface SiO_x_ layer can enhance the connection between Si and C and reduce Si agglomeration [141]. So far, there is no suitable way to adjust the oxygen content in SiO_x_. Low oxygen content cannot guarantee effective suppression of volume change, while high oxygen content will result in a severe capacity drop and a large voltage hysteresis in the voltage curve [142]. Therefore, it is necessary to conduct a controllable and quantitative study of Si surface oxides, which is crucial for the commercial application of Si anode Li−ion batteries.

**Figure 16:**
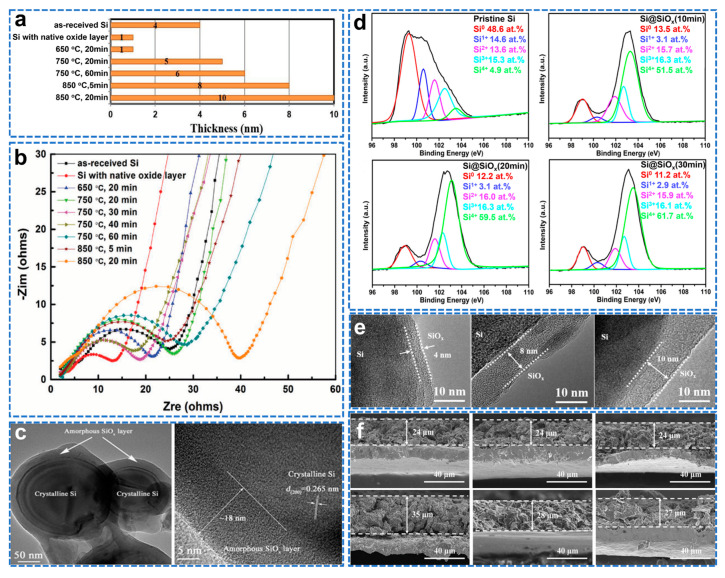
(**a**) Comparison of thickness of the thermally grown oxide layers under different oxidation conditions, (**b**) Electrochemical impedance spectroscopy (EIS) curves after de−lithiation in the second cycle [143]. (**c**) TEM image and HRTEM image for Si@SiO_x_ (20 min) sample, (**d**) High resolution XPS spectra of Si 2p for pristine Si, Si@SiO_x_ (10 min), Si@SiO_x_ (20 min), and Si@SiO_x_ (30 min) samples [144]. (**e**) TEM images of Si−9 h, Si−11 h, and Si−13 h, (**f**) Cross−sectionals before and after 200 cycles of GSC−9 h, GSC−11 h, and GSC−13 h electrodes [145].

Zheng et al. controlled the thickness of the surface oxide to be between 1 and 10 nm by controlling the oxidation temperature and time, as shown in Figure 16a [143]. Through electrochemical experiments and model studies, it is determined that the Si@SiO_x_/C composite anode with a surface oxide layer thickness of about 5 nm has the best electrochemical performance. As the oxidation temperature or time increases, the oxide layer becomes gradually thicker; the samples with a 1 nm thick oxide layer show higher ICE, the samples with a 5 nm thick oxide layer show higher capacity, and the samples with an 8 nm thick oxide layer can achieve better cycling performance at lower capacities, indicating that the oxidation conditions can be tuned to achieve different electrochemical performances to suit different commercialization requirements. As shown in Figure 16b, different oxide layer thicknesses correspond to different resistances. When the surface oxide layer is 6 nm, it has the lowest resistance. The relationship between oxide layer thickness and resistance is not a simple proportional relationship; this requires more in−depth research. Tang et al. used a simple green plasma oxidation strategy to fabricate SiO_x_ layers on the surface of Si nanoparticles [144]. When pristine Si nanoparticles are exposed to plasma conditions, strong oxidizing species are generated between the electrodes and induce surface oxidation of Si nanoparticles, resulting in Si@SiO_x_ material, as shown in Figure 16c. In the experiment, when the exposure time is 20 min and the SiO_x_ layer thickness is 18 nm, the obtained Si@SiO_x_ material has good cycling stability (1201 mAh g^−1^ at 200 cycles) and improves the ICE (89.96%). As shown in Figure 16d, with different exposure times, not only different oxide layer thicknesses can be obtained, but also the structure and valence state of Si in the surface oxides are changed. Si^2+^ and Si^3+^ generally correspond to suboxide; Si^4+^ belongs to silicon oxide, while Si^0^ and Si^1+^ belong to elemental Si, revealing the transition of most elements in the original silicon to silicon oxide/suboxide with increasing exposure time. Zhao et al. designed a dense graphite/silicon/carbon (GSC) composite. As shown in Figure 16e, the thickness of the oxide layer gradually becomes thicker as the sanding time increases [145]. When the sanding time is 11h (GSC−11) and the oxide layer thickness is 8 nm, it has the most stable cycle performance (capacity retention rate reaches 87.3% after 200 cycles at 1C), and the lowest impedance (15.94 Ω). The LiNi_0.5_Co_0.2_Mn_0.3_O_2_/GSC−graphite full cell was assembled and cycled at 1C for 150 cycles, still retaining 1683.5 mAg h^−1^, with a capacity retention rate of 90.1%. When the sanding time is 13 h (GSC−13) and the thickness of the oxide layer is 10 nm, volume expansion is the smallest after 200 cycles, as shown in the Figure 16f. This shows that the larger the thickness of the oxide layer, the smaller the volume expansion, but the excessive thickness of the oxide layer will affect the conductivity of the composite material and affect its electrochemical performance.

In summary, the thickness of the surface oxide layer will affect the electrochemical performance of the silicon anode, but different silicon particle sizes, different oxide layer preparation methods and different subsequent modification methods will affect the oxide layer thickness for the best electrochemical performance, so more in−depth mechanistic studies are needed.

## 5. Conclusions and Perspectives

Si is considered to be the best alternative to graphite anodes due to its higher theoretical capacity, lower discharge potential, and low cost. However, the huge volume expansion of Si during the lithiation process not only leads to material pulverization, but also continuously generates an unstable SEI that continuously consumes the electrolyte. In this review, recent research advances in structural design, oxide complexation and Si alloys are summarized. Second, the effects of surface engineering, pre−lithiation and binders on the electrochemical performance of silicon anode materials are described. Then, the latest characterization techniques for Si—based materials, structural control of Si−graphene composites, SEI formation mechanisms and optimal surface oxide layers are introduced. Finally, the existing problems and future development prospects of silicon−based anode materials are briefly introduced.

Nano−Si increases the contact area of the surface, which can suppress volume expansion well, but its large specific surface area will form more SEI and accelerate the consumption of lithium ions. During the first lithiation process, SiO_x_ reacts to form Li_2_O and Li_4_SiO_4_ as buffer media to form a stable SEI, but its theoretical capacity is lower than that of Si. Alloyed Si has good electrical conductivity, but its low capacity and high cost hinder its commercial application. The conductivity of Si—based materials can be effectively improved by the composite of Si and carbon, thereby further improving the rate capability. The introduction of frameworks such as graphene and MOF can alleviate the volume expansion of Si, avoid direct contact between Si and electrolyte, and form a continuous and stable SEI, thereby improving its electrochemical performance. Table 1 lists typical properties in recent years. However, it is difficult for composites to achieve higher energy densities due to their low Si mass loading. Si and metal−doped composites are difficult to apply on a large scale due to high cost and complex synthesis methods, which hinder their commercial application. At the end of this review, the future development direction of Si anode materials for lithium−ion batteries will be proposed.

(1) SiO_x_ may be more suitable for commercial development: the cycle performance is the bottleneck for the commercial application of Si anode materials. Compared with Si, the volume expansion of SiO_x_ to form inactive products (Li_2_O and Li_4_SiO_4_) during lithiation is greatly reduced, enabling the formation of stable SEI films, thereby improving its cycling performance. Moreover, the reversible capacity of the SiO_x_ electrode is 1500 mAh g^−1^, which is still larger than that of graphite.

(2) Doped Si anodes have better electrochemical performance and cycle stability by improving electrical conductivity, but they usually sacrifice the initial discharge capacity, so they should be comprehensively studied.

(3) Combination of Si—based materials and advanced adhesives are proposed. The functional groups in the polymer adhesives have strong bonding force with the modified Si surface through chemical reaction, which is conducive to the formation of a more stable SEI film, thereby improving the electrochemical performance of the anode. However, more advanced characterization techniques are still needed to observe the relationship between the Si surface, the adhesive and the SEI formation from the microstructure.

(4) Advanced characterization technologies such as advanced in situ time− and space−resolution characterization technologies help to understand the electrochemical mechanism of Si−based anodes during lithiation/de−lithiation, especially the formation and evolution of SEI; these need further study.

(5) From 30 GWH in 2011 to 492 GWH in 2021, higher energy density batteries will be needed in the future. In addition to this, the most difficult aspect of silicon industrialization is the low ICE. Pre−lithiation can substantially improve the ICE of lithium−ion batteries and compensate for irreversible capacity losses. However, the pre−lithiation process is difficult and costly and will be a difficult point for future silicon anode industrialization.

## Figures and Tables

**Table 1:** List of typical properties in recent years.

Anode Materials	Initial Discharge [mAh g^−1^]/Current Density [A g^−1^]	Capacity/Cycles/Current Density [mAh g^−1^]/−/[A g^−1^]	ICE (%)	Ref
Bamboo−like SiO_x_/C	1425/0.1	702/200/0.1	64.6	[146]
porous Si/SiO_x_@C	1437/0.2	1075/350/0.2	66.53	[147]
SiO_x_@C NTs	1373/0.1	713/200/0.1	66.5	[148]
H−SiNS/C	1670/0.1	1040/500/2	55	[149]
C/Si/CNTs	1338.2/01	696.8/50/0.1	54.1	[150]
Si/C−L	2026/0.2	637/40/0.2	76	[151]
Si/SiC/C P−Si/C@C	1907/0.1 1269/0.1	1357/200/0.1 708.6/820/1	78.7 89.8	[152] [153]
Si@SiO_2_@C	2579.8/0.1	1051/100/0.1	84.66	[154]
Si@PPA−7%	/	1316.3/500/2.1	90.1	[155]
P−Si nanoparticles	3610.2/0.1	1645.6/200/0.4	87	[156]
m−Si@NDC	2482/0.2	890/200/4	/	[157]
SiO_x_/C@void@Si/C	1938/0.2	1094/550/0.2	77.85	[158]
SiO_x_/Fe–N–C	1226.9/0.1	799.1/100/0.1	61.8	[45]
SNSC	1732.5/0.2	1577.5/100/0.2	89.3	[159]
PoSi@C−CO_2_	1588/0.4	1124/100/0.4	/	[160]
U−L Si@C@MoS_2_	1391/0.1	1357/250/0.1	80	[161]
MSO@C	1630.9/0.06	688.9/200/0.06	88.7	[162]
SiO_x_@CNT	1012/0.1	1012/500/2	69.3	[64]
C/SiO_x_	3000/0.1	650/1500/1	41.7	[163]
MP Si/C	2222.2/1	671/400/2	78.5	[164]
SnO_y_@C/SiO_x_	500/0.1	530.8/7500/10	46.12	[165]
FSCC PSi@SiO_x_/Nano−Ag PSi/Ag/C SHCM/NCF D−SiO@G SiO_x_@CNT s/C−550 SiO_x_@C@CoO Si@10−ZC L−Si/C−750	1932/0.4 3488/0.5 1800/0.2 2583/0.1 1937/0.1 1289/0.02 1287/0.1 3386/0.2 2609.2/0.2	839/200/0.4 1409/500 1794.6/50/0.2 1442/800/1 740.6/500/2 902/400/1 714/750/1 1741/100/0.2 1473/400/0.2	56.6 86.96 83.33 86 78.2 88 65.7 80 65.89	[166] [70] [167] [30] [168] [16] [169] [170] [28]

## Data Availability

Data are contained within the article.
